# Fabrication of 20.19% Efficient Single-Crystalline Silicon Solar Cell with Inverted Pyramid Microstructure

**DOI:** 10.1186/s11671-018-2502-9

**Published:** 2018-04-03

**Authors:** Chunyang Zhang, Lingzhi Chen, Yingjie Zhu, Zisheng Guan

**Affiliations:** 1 College of Materials Science and Engineering, Nanjing Tech University, Nanjing, 210009 Jiangsu China; 2 Jiangsu Collaborative Innovation Center for Advanced Inorganic Function Composites, Nanjing, 210009 China

**Keywords:** Inverted pyramid, sc-Si solar cell, Metal-assisted chemical etching, Alkaline anisotropic texturing

## Abstract

This paper reports inverted pyramid microstructure-based single-crystalline silicon (sc-Si) solar cell with a conversion efficiency up to 20.19% in standard size of 156.75 × 156.75 mm^2^. The inverted pyramid microstructures were fabricated jointly by metal-assisted chemical etching process (MACE) with ultra-low concentration of silver ions and optimized alkaline anisotropic texturing process. And the inverted pyramid sizes were controlled by changing the parameters in both MACE and alkaline anisotropic texturing. Regarding passivation efficiency, the textured sc-Si with normal reflectivity of 9.2% and inverted pyramid size of 1 μm was used to fabricate solar cells. The best batch of solar cells showed a 0.19% higher of conversion efficiency and a 0.22 mA cm^−2^ improvement in short-circuit current density, and the excellent photoelectric property surpasses that of the same structure solar cell reported before. This technology shows great potential to be an alternative for large-scale production of high efficient sc-Si solar cells in the future.

**Electronic supplementary material:**

The online version of this article (10.1186/s11671-018-2502-9) contains supplementary material, which is available to authorized users.

## Background

Single-crystalline silicon (sc-Si) solar cell has long dominated the solar cell market owing to its high photoelectric conversion efficiency and comprehensive performance [1–5]. However, the advantage of comprehensive quality over other crystalline and noncrystalline silicon solar cell has gradually diminished, due to the rapid development of diamond wire sawing technique, advanced passivation technique, and other type solar cells [6–13]. As reported in practical production, sc-Si solar wafers with upright pyramid structure fabricated in plant production have a mean reflectivity of 10–12%, which almost has reached the limit of one-step alkaline chemical texturing technique [14]. The improvement in photoelectric conversion efficiency gained little from modulation of upright pyramid structure. In order to change this situation, the improvement in conversion efficiency may be probably continued by fabricate new light-trapping structure such as black silicon [15]. The black silicon technique can be used to modify surface with extremely low reflectivity and high light absorption [16]. Due to its ultra-low reflectivity (near 0.3%) in the ultraviolet visible and near infrared region which benefits efficiency improvement, black silicon solar cell has become a very promising direction of conventional sc-Si solar cell [16]. Thus, the conversion efficiency of sc-Si solar cell can be further improved from the perspective of black silicon.

The black silicon technique has immediately become a research hotspot since its discovery in 1995 [17]. There are three dominant techniques based on nanostructure fabrication: femtosecond laser technique, reactive ion etching (RIE), and metal-assisted chemical etching (MACE) [16, 18, 19]. Given the compatibility of current sc-Si solar cell technology and cost, MACE is the optimal solution to replace conventional alkaline texturing technology [20]. The great light-trapping ability of MACE-fabricated black silicon is beneficial to improve photoelectric conversion efficiency of sc-Si solar cells. However, a lower reflectivity of black silicon corresponds to more nanostructures, which would enlarge surface defect area and accelerate indirect recombination of photo-generated carriers, thereby restraining the photoelectric conversion efficiency [21].

Many pertinent works have been done to solve the problem above. Specifically, the conversion efficiency of sc-Si solar cell can be enhanced by either optimizing the surface structure for light trapping or improving the passivation technique [20, 22]. Savin et al. introduced atomic layer deposition (ALD) into the passivation process and combined it with the interdigitated back contact crystalline silicon solar cells, and the solar cell conversion efficiency reached 22.1% [23]. Despite the improvement of conversion efficiency, however, the application into large-scale industrial production was still limited by despairing costs. RIE-fabricated black silicon could significantly increase light-trapping ability, but the investment in hardware equipments was large which made it hard to be applied in mass production or less competitive against wet chemical texturing technology. The inverted pyramid structure obtained low surface area and great light-absorbing ability [24–26]. Stapf et al. used mixed solution of hydrogen peroxide (H_2_O_2_), hydrofluoric acid (HF), and hydrochloric acid (HCl) to texture sc-Si, and random inverted pyramid structures were accessed, but the light-trapping ability of inverted pyramid structure was still under investigation [27]. The mechanism of MACE (metal = Au, Cu, and Fe) has been explored, and its application in crystalline silicon surface texturation is also studied [28–34]. However, the concentrations of metal ions in MACE ever reported, applied for crystalline silicon solar cells, were very high, which disobeyed the increasingly harder environmental protection policies and cost too much. Moreover, the texturation fabricated in MACE reported before was mostly explored to generate nanostructures as much as possible for light-absorbing ability rather than practical application. It was rarely reported about black silicon technique with low cost, which obtained potential in plant production. Our team introduced MACE with Ag nanoparticles into sc-Si texturing process at a low cost and optimize the MACE process by using specific etching additive, which reduced the concentration of Ag ion to two orders-of-magnitude lower than ever reported [32]. Furthermore, the required temperature of alkaline anisotropic texturing process was relatively lower than that in industrial production.

In this work, the optimized MACE technique was introduced into post rinse treatment of sc-Si solar cell, which promoted the photoelectric performance. Black silicon solar cells with inverted pyramid structure manufactured in bulk were accessed, which the conversion efficiency was up to 20.19%. Meanwhile, the formation mechanism of inverted pyramid structure was studied. As expected, black silicon solar cell with inverted pyramid microstructure showed a great potential in large-scale industrial production.

## Methods

Diamond wire sawing (100)-oriented P-type sc-Si wafers (200 ± 20 μm thick, 1–3 Ω cm) with standard solar cell size of 156.75 × 156.75 mm^2^ were used in this experiment. The wafers were rinsed in an aqueous solution consisting of NaOH (AR) and H_2_O_2_ (30 wt.%) to remove surface impurities and then rinsed in ultra-pure water. In the MACE process, firstly, wafers were immersed in an aqueous solution containing HF (0.2 M) and AgNO_3_ (3 × 10^−5^ M) at 25 °C. Then, nanoporous silicon structures were fabricated when the silicon wafers coated with Ag nanoparticles were etched in the mixed acid solution of H_2_O_2_ (3.13 M) and HF (2.46 M) for 3 min, which contained 0.1% commercial additive (C, Nanjing Natural Mew Material Co. Ltd., China). The wafers with nanoporous structures were rinsed in ammonia water (0.1 M) with H_2_O_2_ (0.1 M) for 5 min to remove residual Ag nanoparticles. After being rinsed in ultra-pure water, nanoporous silicon structures were modified in an aqueous solution of NaOH (0.003 M) and 0.4% commercial additive (A, Nanjing Natural Mew Material Co. Ltd., China) at 60 °C. Finally, the industrial process for sc-Si solar cells was to produce inverted pyramid solar cells. The detailed steps were phosphorus element diffusion to form p-n junction emitters, acid etching to remove phospho silicate glass, plasma-enhanced chemical vapor deposition (PECVD) to deposit SiNx antireflection layer, and screen printing to metallize bottom/top electrodes.

The sc-Si surface morphology was observed under cold field emission scanning electron microscope (SEM; Hitachi S-4800, Japan). The sizes of sc-Si surface microstructure were measured on a Zeta 3D metrology system. The optical reflectance index from 300 to 1000 nm was measured by a UV-VIS and NIR spectrophotometer (UV-3101PC, Japan, with an integrating sphere). The SiNx film was measured by film thickness measurement system (Filmetrics, F20-UV, USA). The internal/external quantum efficiency and photovoltaic conversion efficiency of sc-Si solar cells were measured by Enlitech QE-R and PVIV-411V systems, respectively.

## Results and Discussion

**Fig. 1:**
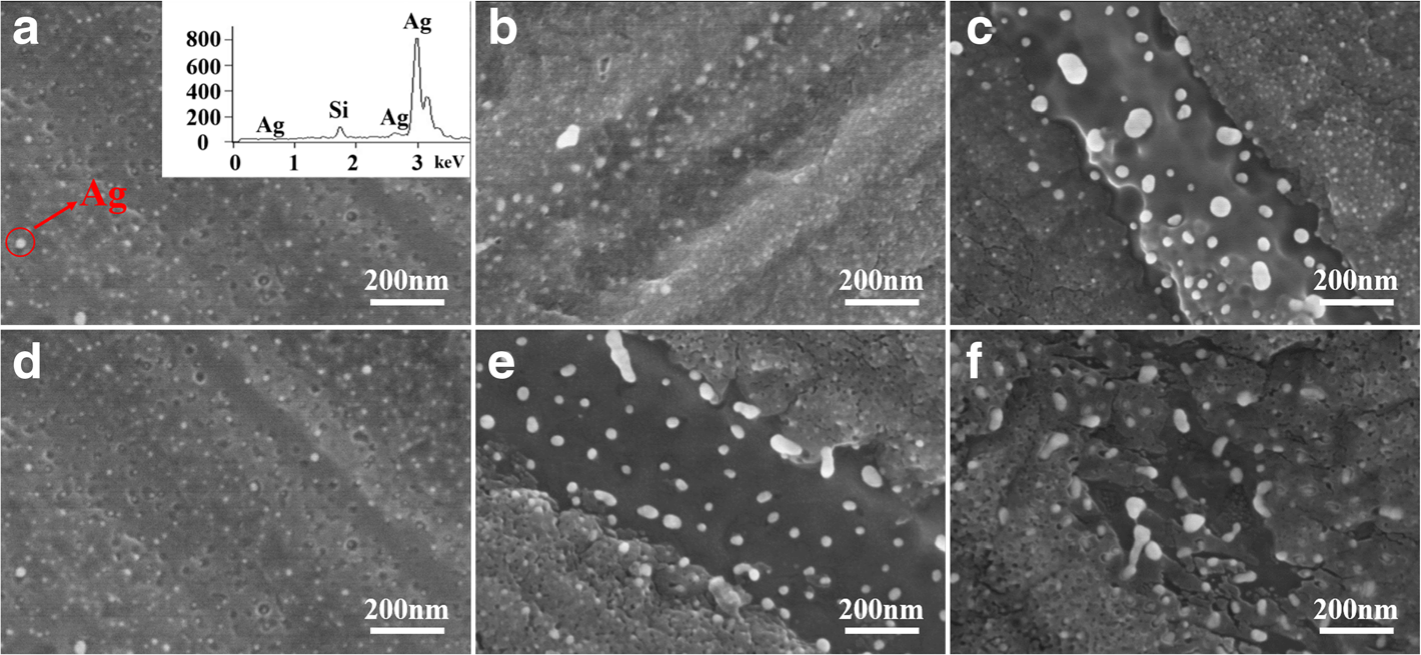
SEM images of Ag nanoparticles deposited on sc-Si and insert of EDS. SEM images of Ag nanoparticles deposited on sc-Si: **a**–**c** deposition for 2 min at 25 °C with Ag ion concentration of 5, 10, and 15 ppm, respectively; and **d**–**f** deposition at 25 °C with 5 ppm concentration Ag ion for 2, 4, and 6 min, respectively. EDS result in the inset of **a**

As reported previously, electroless metal nanoparticles deposited on Si in aqueous solution containing HF were well investigated before [35]. The electroless Ag nanoparticle deposition used in MACE was based on the galvanic displacement reaction while two electrochemical processes occurred simultaneously around the sc-Si surface [36]. SEM images in Fig. 1a–f show the Ag nanoparticles deposited on p-type (100)-oriented sc-Si surface by immersion in an HF solution containing AgNO_3_. As shown in Fig. 1a–c, Ag nanoparticles were fabricated into the sc-Si surface in the aqueous HF solution containing 5 to 15 ppm AgNO_3_ at 25 °C for 2 min.

Figure 1a clearly shows that white sediment was deposited into the sc-Si substrate, which was verified by an energy-dispersive spectrometer (EDS: inset in Fig. 1a) to be Ag nanoparticles. The reduced Ag nanoparticles substituted silicon where the oxidizing reaction happened and deposited on the silicon substrate. Ag nanoparticles of 15 nm in diameter were distributed evenly and densely with the presence of 5 ppm AgNO_3_ (Fig. 1a). However, with 10 ppm AgNO_3_ or higher concentration, the diameters of Ag nanoparticles increased unevenly (Fig. 1b, c). The diameter of regional Ag nanoparticles in Fig. 1b increased to 80 nm, and that in Fig. 1c was up to 100 nm. SEM images in Fig. 1d–f show the Ag nanoparticles deposited for 2, 4, and 6 min, respectively, at which was 5 ppm AgNO_3_ and 25 °C. It illustrates that shape of Ag sediment changed much and became irregular (varied from one dimension to two dimensions) with deposition time being prolonged. Moreover, these stick-shaped Ag nanoparticles (around 130 nm in length) deposited on the sc-Si surface irregularly by time delaying, which destroyed the uniformity of Ag nanoparticle distribution. In summary, we propose the Ag ion concentration at 5 ppm and deposition time for 2 min at room temperature.

**Fig. 2:**
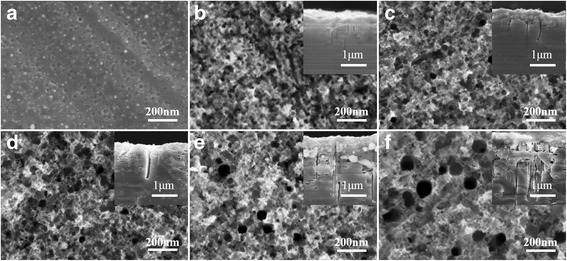
SEM images of nanoporous silicon (cross section in insert) with different processing time. SEM images of nanoporous silicon: **a** as-fabricated and **b**–**f** nanoporous silicon and cross section in inset for 1, 2, 3, 4, and 5 min processing at 35 °C

**Fig. 3:**
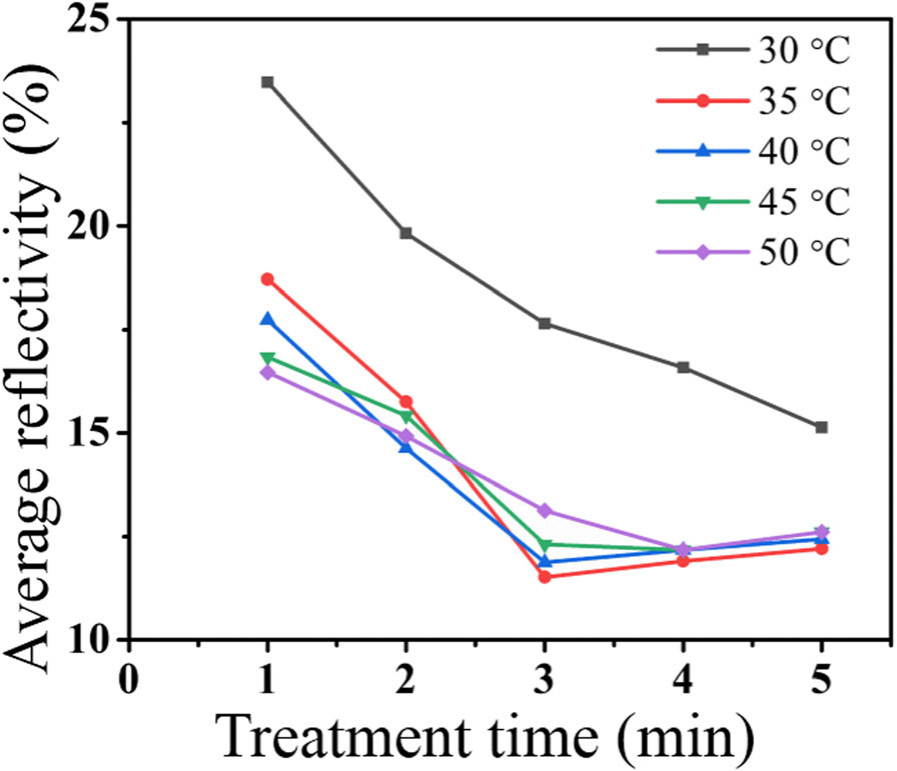
Average reflectivity nanoporous silicon dependence of time at specific temperatures. Average reflectivity of nanoporous silicon structures dependence of time for treatment at 30, 35, 40, 45, and 50 °C, respectively

The sc-Si wafers with uniform Ag nanoparticles coating were immersed in mixed acid solution containing commercial additive to fabricate nanoporous silicon structure. This commercial additive that might be a mixture of polyol containing hydroxyl and carboxyl was to separate minute bubbles from the substrate surface because H_2_ generated in the reaction could not get away from substrate surface automatically in the case of such low Ag concentration (Additional file 1). SEM images in Fig. 2a–f show the morphologies of nanoporous silicon and cross section before and after MACE. As shown in Fig. 2b, nanoporous silicon structures generated in the sc-Si with MACE processing for 1 min. The diameter of nanoporous silicon reached to 20 nm and depth about 1.3 μm. Then, diameter and depth of nanoporous silicon both increased with the prolonging of MACE time, even the diameter varied more obviously. Diameter of nanoporous silicon with MACE processing for 2 min grew to 40 nm, then 50 nm for processing 3 min, 80 nm for processing 4 min, and 110 nm for processing 5 min. Cross section insets in Fig. 2b–f show the depth of nanoporous silicon varied from 1.3 to 3 μm when MACE time increased from 1 to 5 min. However, quite a few nanoholes in cross section generated when MACE time was prolonged. According to Chartier’s report, nanoporous silicon generated in MACE included straight and curved cylindrical pore structures, and the straight nanoholes dominate when the etching solution molar ratio *ρ* = [HF]/([HF] + [H_2_O_2_]) is about 45% [36]. Despite the *ρ* = 45% in our work, a large amount of curved cylindrical pores generated with over-time etching when MACE processed for 4 min or more (cross sections in both insets of Fig. 2e, f). Through series of experiments, we observed that light-trapping ability of nanoporous silicon decreased with over-time MACE processing. The average reflectivity of nanoporous silicon against MACE treatment time at different temperatures is illustrated in Fig. 3. The average reflectivity minimized for MACE processing 3 min at 35 °C and then increased with time delaying. Meanwhile, the average reflectivity of nanoporous silicon changed little when temperature was 35 °C or higher. It could be explained by the fact that generation of curved cylindrical nanoholes made the nanoporous silicon structures hollow and messy instead of vertical, then incident light might be reflected back to the air through those curved nanoholes. On the other hand, nanoporous silicon itself was oxidized and dissolved slowly in mixed solutions of HF and H_2_O_2_ which made the substrate surface smooth and the average reflectivity increased. Similar tendency of reflectivity changing when temperature was above 30 °C showed that the convenient MACE temperature was at 35 °C. In conclusion, nanoporous silicon structures were fabricated in MACE process with ultra-low concentration of Ag ion, which was never reported before. The optimizing condition (temperature at 35 °C and time for 3 min) in MACE to fabricate vertical nanoporous silicon structure is proposed.

**Fig. 4:**
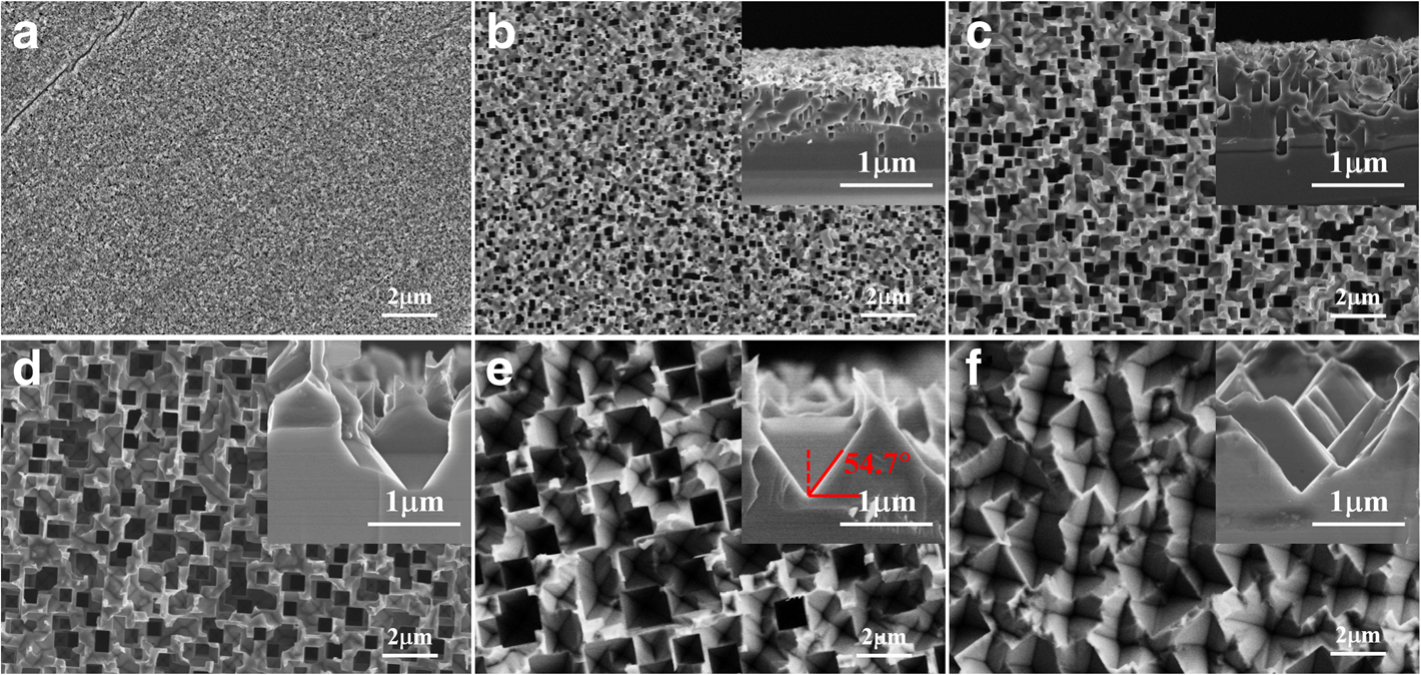
SEM images of inverted pyramid (cross sections in insert) processing for different time. SEM images: **a** nanoporous silicon and **b**–**f** inverted pyramid surface-section and cross section texturing at 60 °C in aqueous NaOH solution for 1, 3, 5, 7, and 9 min, respectively

**Fig. 5:**
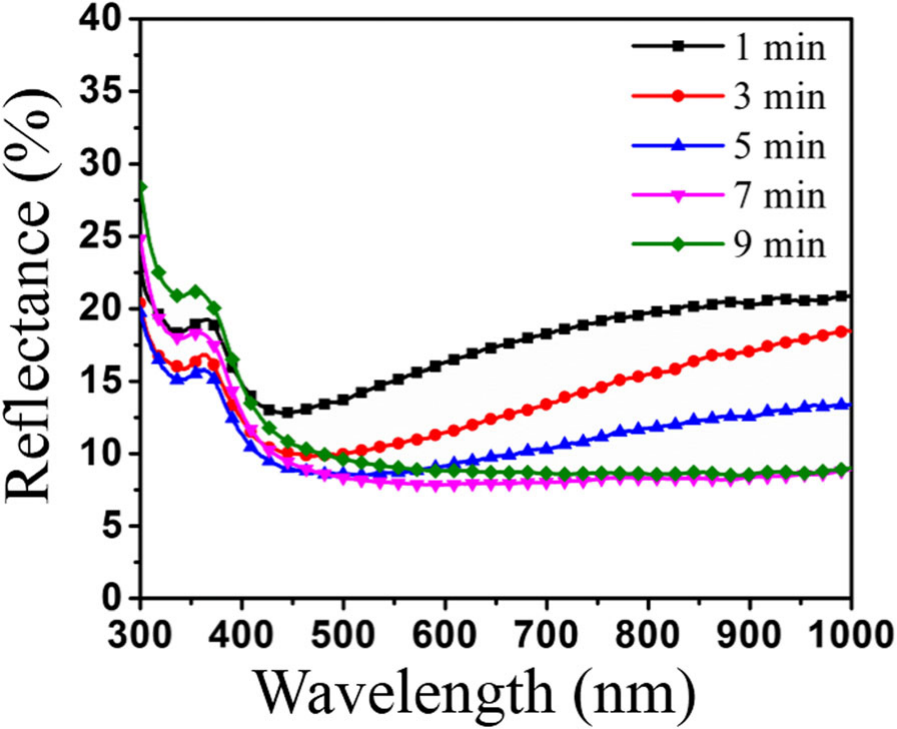
Reflectance spectra of inverted pyramid structures with texturing for different time. Reflectance spectra of sc-Si with inverted pyramid structures for alkaline texturing time at 1, 3, 5, 7, and 9 min, respectively

Nanoporous silicon generated by MACE underlies the formation of inverted pyramid structures. The wafers were modified in the alkaline anisotropic texturing process and additive A in NaOH aqueous solution played a similar role like surfactants in conventional sc-Si texturation. It removed bubbles from the substrate surface and influences the anisotropic factor of etchant. Finally, inverted pyramid structures were accessed. Figure 4a shows the nanoporous silicon structure, and Fig. 4b–f shows inverted pyramid structures with NaOH texturing for 1, 3, 5, 7, and 9 min, respectively. Figure 4b, c shows the nanoporous silicon structures turned into square holes with inverted pyramid-shaped bottom (inset in Fig. 4b, c) with alkaline anisotropic processing for 1 and 3 min, respectively. With texturing time being prolonged, the inverted pyramid structures were growing as shown in Fig. 4c–f, and specific areas were dissolved gradually. When the alkaline chemical texturing treated for 5 min, the inverted pyramid structures with 500 nm in width and 350 nm in depth were fabricated. However, there existed quite a few defect structures (inset of Fig. 4d). As shown in Fig. 4e, inverted pyramids in width of 1 μm were fabricated and distributed uniformly when texturing processed for 7 min. The dihedral angle was 54.7° and less defect structures existed observed from the cross section (inset in Fig. 4e). When the treatment time was up to 9 min, the inverted pyramids had smooth surface and seldom defect structures (Fig. 4f). However, it was easily observed that some side walls of inverted pyramids were dissolved, and new micro-scale gully arrays with size varied from 2 to 4 μm were formed. The dissolution of side walls made the overlapped structures generated (inset in Fig. 4f). Despite the fact that inverted pyramid structures were distributed with hardly no defect areas, large pit structures might decrease the light absorbance ability. Figure 5 shows the reflectance spectra of inverted pyramid structures with alkaline anisotropic texturing for 1, 3, 5, 7, and 9 min, respectively. The reflectance spectra showed that light-trapping ability decreased compared with original nanoporous silicon due to large quantities of nanostructure dissolution when alkaline texturing processed for 1 min. The average reflectivity in wavelength range from 300 to 1000 nm is 15.45%. Clearly, with texturing time increasing, light absorbance was enhanced gradually for the formation of inverted pyramid structures. The reflectivity minimized to 9.2% when texturing processed for 7 min, and the uniformity of inverted pyramid sc-Si wafers reached to best compared with others. Then light-trapping ability decreased, and reflectivity rose up to 10.5% with texturing for 9 min, caused by dissolution of inverted pyramids and formation of large-size overlapped pit structures. What is more, this sc-Si texturation was more reflective than that in plant production. Thus, nanoporous silicon structures were textured in aqueous NaOH solution containing specific compound additive, and uniformly distributed inverted pyramid structures with 1 μm size in width were accessed at 60 °C for 7 min. The average reflectivity was controlled at 9.2%.

**Fig. 6:**
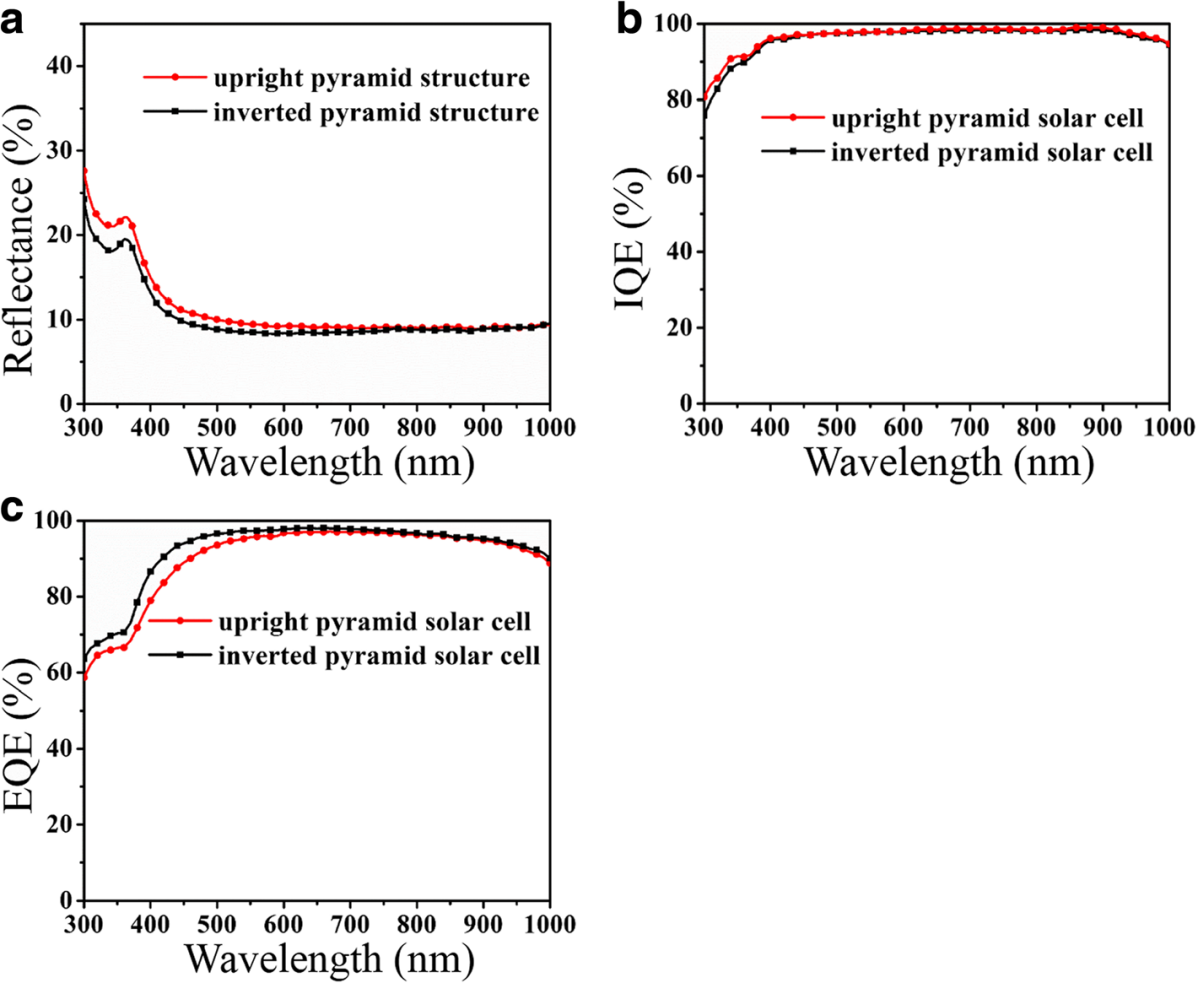
Comparison of **a** reflectance spectra, **b** IQE, and **c** EQE. **a** Reflectance spectra of inverted and upright pyramid structures. **b** IQE and **c** EQE of inverted and upright pyramid sc-Si solar cells

Considering both light-trapping ability and easy design of surface microstructure for passivation, we chose the inverted pyramid structure with width of 1 μm to fabricate solar cells. Box resistance and SiNx film property by PECVD of inverted and upright pyramid sc-Si wafers are compared in Table 1. We tested ten sets of the test samples and control samples (each set contained 10 pieces). The gap of average box resistance between inverted and upright pyramid sc-Si wafers was small, even the uniformity of inverted pyramid sc-Si distribution led that of upright one a little observed from the STD data. Comparison of SiNx film passivation property by PECVD suggests the SiNx film passivated on sc-Si with inverted pyramid structure is 10 nm thinner and has a refractive index 0.14 higher compared with upright pyramid. It means that passivation cost of inverted pyramid structure might be lower than upright one especially when the SiNx film property passivated on inverted pyramid structure is similar to that of upright one. It is beneficial to the industrialization application of this texturing technology. The average reflectivity, internal quantum efficiency (IQE), and external quantum efficiency (EQE) are shown in Fig. 6. The average reflectivity of inverted pyramid structure in width with 1 μm was 1% lower than that of upright ones in plant production (Fig. 6a). The SiNx film deposition process of sc-Si solar cell with inverted pyramid structure was the same as that of upright pyramid sc-Si. As shown in Fig. 6b, IQE of inverted pyramid sc-Si solar cell was similar to that of upright one. In the other hand, EQE of sc-Si solar cell with inverted pyramid structure shown in Fig. 6c got improved in wavelength 300–600 nm. It was assumed that unoptimized PECVD technique impeded the improvement of IQE of inverted pyramid sc-Si solar cell, and the lead of EQE in short wavelength from 300 to 600 nm might be attributed to reflectivity superiority in short wavelength described above.

**Table 1:** Comparison of box resistance in P-N injection and PECVD effect between inverted and upright pyramid structures

	Box resistance in P-N injection		PECVD	
	Average/Ω	STD	Thickness of SiNx film/nm	Refractive index
Inverted pyramid sc-Si	80.44	4.36	73.05	2.00
Upright pyramid sc-Si	80.32	4.48	83.75	1.96

**Fig. 7:**
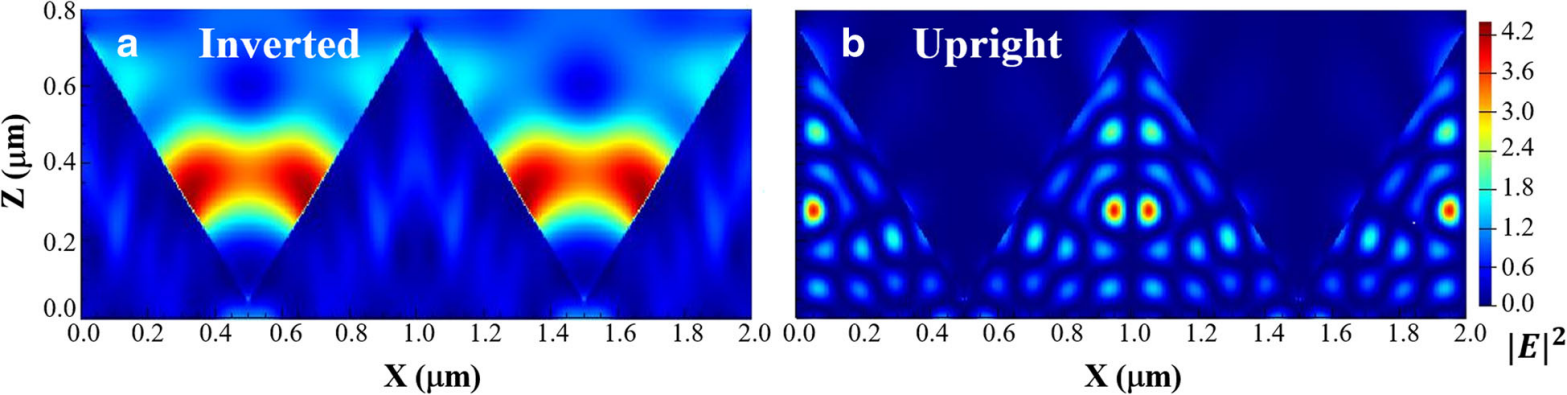
FDTD simulation of electric field intensity distribution in inverted/upright pyramid structure sc-Si. 3D FDTD simulation of electric field intensity distribution in inverted/upright pyramid structure sc-Si. Inverted/upright pyramid size are designed at 1 μm

Three-dimensional (3D) finite difference time domain (FDTD) analysis was used to simulate and analyze photovoltaic effect near the interface of inverted pyramid structure. The simulation dimension of inverted/upright pyramids was designed at 1 μm in width. We used *λ* = 631.57 nm to calculate the electrical field intensity (|E|^2^) distribution of electromagnetic wave, which was close to the peak irradiance of solar spectra. As the simulation results shown in Fig. 7a, b, the energy of electromagnetic wave at 631.57 nm mostly gathered inside of inverted pyramid, which was much stronger than that of upright one. This simulation finding confirms the stronger photon-capturing ability of inverted pyramid structure.

The main electrical performance comparison of two types of sc-Si solar cells are shown in Table 2. The sc-Si solar cell with inverted pyramid structure shows a higher efficiency of 20.19% and short-circuit current density (*J*_sc_) 0.22 mA cm^−2^ higher than that of upright one, which reconfirms the 3D FDTD simulating finding. The open-circuit voltage (*V*_oc_) of sc-Si solar cell with inverted pyramid structure reached to 647 mV, which was 2 mV higher than that of upright pyramid solar cell. In combination with IQE result, *V*_oc_ advantage of inverted pyramid solar cell would be expanded if passivation technique was optimized. Its filling factor (FF) was 0.05% higher than the upright one. Further measures of improvement in photoelectric conversion efficiency should be focused on effective restriction of Auger recombination, stronger light-trapping ability, and better passivation technique.

**Table 2:** Comparison of electrical properties of inverted and upright pyramid sc-Si solar cells

	*V*_oc_/mV	*J*_sc_/mA cm^−2^	R_s_/Ω cm^2^	FF/%	Eff./%
Upright pyramid Si	645	38.25	0.003425	80.87	20.0
Inverted pyramid Si	647	38.47	0.003522	80.92	20.19

## Conclusions

In summary, the sc-Si with inverted pyramid microstructure fabricated by modulated alkaline texturing combined with optimized MACE showed great potential in optimizing both optical reflectivity and microstructure size compared with any other texturing technologies. The conversion efficiency of sc-Si solar cells with inverted pyramid structure designed with the size of 1 μm reached 20.19%, and short-circuit current density of solar cell was up to 38.47 mA cm^−2^. Predictably, cell property will be improved if the optimization of inverted structure or texturation technology continues.

## Additional file

Additional file 1: **Figure S1.** Morphology comparison of silicon wafers processed in MACE with and without additive C: (a) with additive C, front view; (b) without additive C, front view; (c) without additive C, oblique view. (DOCX 1014 kb)

## Abbreviations

3D: Three-dimensional
ALD: Atomic layer deposition
EDS: Energy-dispersive spectrometer
EQE: External quantum efficiency
FDTD: Finite difference time domain
FF: Filling factor
IQE: Internal quantum efficiency
*J*_sc_: Short-circuit current density
MACE: Metal-assisted chemical etching
mc-Si: Multi-crystalline silicon
PECVD: Plasma-enhanced chemical vapor deposition
RIE: Reactive ion etching
sc-Si: Single-crystalline silicon
SEM: Scanning electron microscope
STD: Standard deviation
*V*_oc_: Open-circuit voltage
