# Supplementary material for: Fabrication of 20.19% Efficient Single-Crystalline Silicon Solar Cell with Inverted Pyramid Microstructure
Source: Nanoscale Res Lett. 2018 Apr 3;13:91. doi: 10.1186/s11671-018-2502-9 (PMC5882476; doi:10.1186/s11671-018-2502-9)
Supplement: Supplementary file 1 — Figure S1. Morphology comparison of silicon wafers processed in MACE with and without additive C: (a) with additive C, front view; (b) without additive C, front view; (c) without additive C, oblique view. (DOCX 1014 kb) [file 11671_2018_2502_MOESM1_ESM.docx]

Additional file 1

**Fabrication of 20.19% efficient single-crystalline silicon solar cell with inverted pyramid microstructure**

Chunyang Zhang^1, 2^, Lingzhi Chen^1, 2^, Yingjie Zhu^1^ and Zisheng Guan^1, 2^

^1^ College of Materials Science and Engineering, Nanjing Tech University, Nanjing, Jiangsu 210009, China

^2^ Jiangsu Collaborative Innovation Center for Advanced Inorganic Function Composites, Nanjing, 210009, China

E-mail address: Chunyang Zhang: llyaomo@njtech.edu.cn

Lingzhi Chen: scdzclz@njtech.edu.cn

Yingjie Zhu: sakuno@njtech.edu.cn

Corresponding author: Zisheng guan: zsguan@njtech.edu.cn


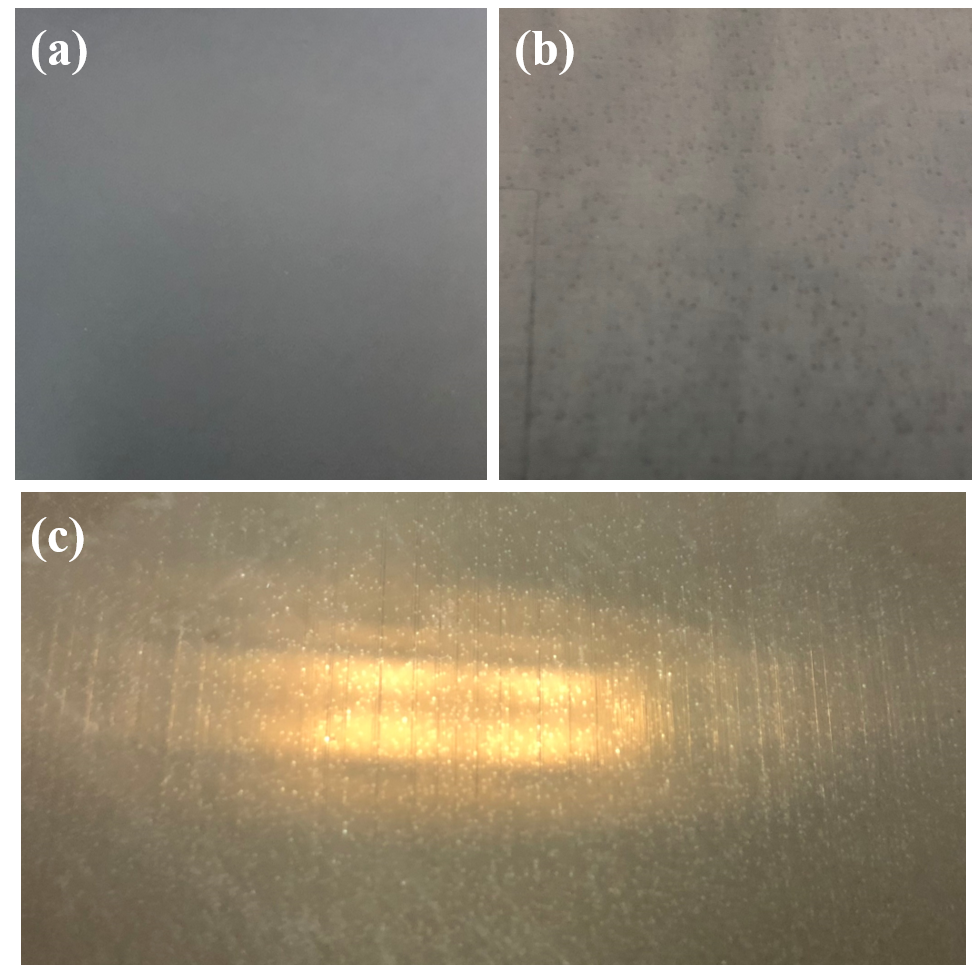


**Figure S1** Morphology comparison of silicon wafers processed in MACE with and without additive C: (a) with additive C, front view; (b) without additive C, front view; (c) without additive C, oblique view.
